# Concurrent Validity of the Japanese Version of the International Physical Activity Questionnaire Long Form for Assessing Walking Behavior Across Adulthood: Applicability to Older Adults

**DOI:** 10.7759/cureus.81328

**Published:** 2025-03-28

**Authors:** Hiroko Shimura, Shinpei Okada, Kaori Daimaru, Naoki Deguchi, Yoshinori Fujiwara, Hiroyuki Sasai

**Affiliations:** 1 Research Team for Promoting Independence and Mental Health, Tokyo Metropolitan Institute for Geriatrics and Gerontology, Tokyo, JPN; 2 Research Department, Physical Education and Medicine Research Foundation, Nagano, JPN; 3 Research on Social and Human Sciences, Tokyo Metropolitan Institute for Geriatrics and Gerontology, Tokyo, JPN

**Keywords:** accelerometer, adults, international physical activity questionnaire, physical activity, validity, walking behavior

## Abstract

Introduction: Walking behavior may vary across age groups, highlighting the need for a validated tool to assess walking including older adults. While the Japanese version of the International Physical Activity Questionnaire long form (Japanese IPAQ-L) has been validated for total physical activity in younger populations, its validity in assessing walking behavior remains unclear in any age group.

Objective: This study aimed to evaluate the concurrent validity of the Japanese IPAQ-L for assessing walking behavior throughout adulthood, with a special focus on its applicability to older adults.

Methods: Participants wore an ActiGraph wGT3X-BT (ActiGraph LLC, Pensacola, Florida, United States), an accelerometer-based activity monitor, around their waist for seven consecutive days to objectively measure their physical activity. On the following day, they completed the Japanese IPAQ-L, which asked about their domain-specific physical activity over the preceding seven days. Spearman's rank correlation coefficients (ρ) were calculated to examine the associations of walking (total, work, transport, leisure) and moderate physical activity, both subjectively assessed by the Japanese IPAQ-L, with objectively measured moderate physical activity (3.00-5.99 metabolic equivalents) by the ActiGraph.

Results: All 130 recruited participants completed the data collection. Of these, 113 participants without missing data (aged 23-89 years; 51.3% women) were included in the analysis. Self-reported total walking showed weak to moderate correlations with objectively measured moderate physical activity across all age groups, with the strongest correlation observed in the oldest age group (ρ=0.32 for the total group; ρ=0.38 for the youngest age group; ρ=0.35 for the middle-aged group; ρ=0.55 for the oldest age group). For domain-specific walking, the correlations varied by age group. Meanwhile, no meaningful correlations were found for self-reported moderate physical activity, regardless of whether walking was included.

Conclusions: The Japanese IPAQ-L showed weak to moderate concurrent validity for assessing total walking when compared to accelerometer-based activity data across age groups. Notably, the validity was higher in older adults. These findings suggest that the Japanese IPAQ-L can be used to rank individuals based on their total walking, with reasonable applicability to older adults.

## Introduction

Walking is a popular and widely accessible form of physical activity that offers extensive health benefits, from improving cardiovascular health to enhancing mental well-being [1,2]. As one of the most common physical activities [3,4], walking constitutes a substantial portion of adults' overall physical activity [5] and is central to public health strategies aimed at promoting active lifestyles [6,7]. Walking generally corresponds to moderate physical activity [8,9], making it a key contributor to meeting physical activity guidelines [10]. Its simplicity and low barrier to participation make walking an important focus for interventions across all age groups, including older adults, for whom maintaining mobility is crucial for independent living and overall well-being.

Understanding walking behavior is essential for developing effective intervention strategies to promote active lifestyles and improve population health. Evaluating walking behavior across diverse populations is key to identifying patterns and exploring associated health implications. The International Physical Activity Questionnaire (IPAQ) [11] is among the most widely used tools for estimating physical activity behavior, and it enables the evaluation of walking separately from moderate and vigorous physical activity. Its widespread use can be attributed to its standardized format, suitability for large-scale epidemiological surveys, and availability in multiple languages through validated translations [12].

The IPAQ was originally developed and validated for adults aged 18-65 years across 12 countries [11]. However, given the growing emphasis on active aging and the increasing proportion of older adults in many countries [13], it is important to assess its applicability beyond the originally targeted age range. The IPAQ short form (seven items) is recommended for national monitoring, providing a broad overview of overall physical activity, walking, and sitting behavior. However, it does not distinguish between different domains of physical activity. In contrast, the IPAQ long form (27 items) offers a more detailed assessment by capturing physical activity and walking behaviors across four distinct domains, namely, work, transport, domestic, and leisure-time physical activity, while also evaluating overall sitting behavior. The long form allows for a more detailed understanding of walking behavior, which is particularly relevant when considering age-related differences in activity patterns.

Regarding walking, previous studies have reported a significant correlation with objectively measured moderate physical activity, using several language versions of IPAQ, both in the short form [14] and in the long form [15], targeting middle-aged and young to older adults, respectively. For the Japanese version, a significant correlation has been observed between walking behavior assessed by the short form and objectively measured moderate physical activity in older adults [16]. Although the validity of total physical activity in the Japanese version of the IPAQ long form (Japanese IPAQ-L) has been established in relatively younger populations [17], the validity of its walking assessment remains unclear in any age group.

To address this gap, the present study aimed to evaluate the concurrent validity of the Japanese IPAQ-L for assessing walking behavior across a broad range of ages, from young to older adults. By comparing self-reported walking behavior with objectively measured moderate physical activity, this study sought to examine whether the Japanese IPAQ-L provides a valid assessment of walking behavior for all age groups, including older populations. Establishing the validity of the Japanese IPAQ-L could enhance public health strategies and improve physical activity monitoring in this population.

## Materials and methods

Participants and ethics approval

A convenience sample was recruited from communities across several prefectures in Japan, including the National Capital Region (Tokyo, Kanagawa, Saitama, and Yamanashi) as well as other regions (Nagano and Gifu). We sampled participants to achieve, as closely as possible, an equal number of individuals in each 10-year age group (20s, 30s, 40s, 50s, 60s, 70s, and 80s), with the number of participants per 10-year age group ranging from 13 to 23. Eligibility criteria included being between 20 and 89 years of age, having no gait abnormalities, and being willing to wear activity monitors during the data collection period. Adults aged 70 years or older were recruited from the participants of the 2022 Itabashi Longitudinal Study on Aging, an observational study involving comprehensive health examinations and postal surveys of older adults living in Itabashi Ward, Tokyo [18], with an additional criterion of having no significant cognitive impairment.

Participants who attended the introductory session were given a full explanation of the purpose and potential risks of the study. They signed an institutionally approved informed consent form prior to the start of the data collection period. This study was approved by the Institutional Review Board of the Tokyo Metropolitan Institute for Geriatrics and Gerontology (approval number: R22-013).

Data collection and processing

Procedure

Data were collected over approximately one week between January and May 2023, with the specific data collection period varying by participant. Participants self-reported their sociodemographic and anthropometric information in the introductory session (day 0). Behavioral data were collected using the ActiGraph wGT3X-BT (ActiGraph LLC, Pensacola, Florida, United States) activity monitor (days 1-7) and the Japanese IPAQ-L [17] (day 8), both of which were returned via postal mail, using a prepaid return envelope. The ActiGraph and the Japanese IPAQ-L were distributed in the introductory session, where participants also received instructions on how to wear the ActiGraph and how to complete the Japanese IPAQ-L.

Participants received a ¥5,000 gift card as compensation for their time and effort in the study. This compensation was provided regardless of the amount of data collected to ensure voluntary participation.

Sociodemographic and Anthropometric Information

Participants reported their age, gender, living arrangement ("living alone" or not), work status ("work full-time", "work part-time", or "not working"), and perceived health (five-point Likert scale ranging from "poor" to "excellent"). Weight and height were self-reported and used to calculate body mass index (BMI), which was then categorized as either normal (<25 kg/m^2^) or overweight/obese (≥25 kg/m^2^).

Walking and Physical Activity Behaviors

Japanese IPAQ-L: Participants filled out the Japanese IPAQ-L (self-administered format for the last seven days) [17] on day 8, after completing the seven-day period of activity monitor wear, as will be described later. The questionnaire included 27 items assessing physical activity and sedentary behavior across five domains: work, transportation, housework, leisure, and sedentary time. In this study, the main behaviors examined were walking at work, for transport, for leisure, total walking (the housework domain does not include questions about walking), and total moderate physical activity.

To assess walking for transport, participants were asked: "During the last seven days, on how many days did you walk for at least 10 minutes at a time to go from place to place?" (number of days) and "How much time did you usually spend on one of those days walking from place to place?" (hours and minutes). Similar questions were posed regarding walking "as part of your work" (if applicable) and "in your leisure time" to determine walking at work and for leisure, respectively. For moderate physical activity, participants were asked about their physical activity at work, during household chores, and during leisure time. Importantly, moderate physical activity was assessed separately from walking. The Japanese IPAQ-L data were cleaned and processed in accordance with the IPAQ Scoring Protocol [19].

ActiGraph activity monitor: Objective behavioral data were collected over seven consecutive days (days 1-7), using ActiGraph activity monitors under free-living settings. Participants were instructed to wear an elastic belt around their waist with the device positioned on the left side during waking hours, except when engaging in water-based activities (e.g., showering, bathing, or swimming) or playing contact sports. Although the participants did not receive reminders or adherence checks during the seven-day data collection period, they were informed that they could contact the research team by phone at any time if they had questions or concerns. The ActiLife software (Version 6.13.4, ActiGraph LLC) was used to initialize the devices, download the data, and convert raw acceleration data to one-minute epoch activity count data.

Non-wear time was defined as periods of at least 90 consecutive minutes with zero counts (window 1), allowing for short intervals of up to two minutes with nonzero counts (allowance interval), provided there were zero counts in the 30-minute upstream and downstream windows (window 2). Any nonzero counts outside this allowance were considered as wearing [20,21]. In this study, non-wear time was determined using the PhysicalActivity package in R (R Foundation for Statistical Computing, Vienna, Austria (https://www.R-project.org/)) [21].

After excluding non-wear time, daily time spent in moderate physical activity (3.00-5.99 metabolic equivalents, 1952-5724 vertical axis activity counts/min) [22] was aggregated for each participant. Days with 10 hours or more of wear time were considered valid for analysis (convention for compliant wear time) [23]. The valid days were used to calculate the total minutes per week spent in moderate physical activity.

Statistical analysis

Sociodemographic and anthropometric characteristics were summarized using frequencies and percentages for categorical variables. Behavioral variables, assessed using the Japanese IPAQ-L and ActiGraph, were summarized as medians and interquartile ranges. Spearman's rank correlation coefficient (ρ) was used to evaluate the strength of the relationships between the Japanese IPAQ-L and ActiGraph data, assessing the concurrent validity of the Japanese IPAQ-L. Analyses were conducted both for the total sample and by age group. Correlation coefficients were interpreted using the following criteria: negligible (0.00-0.10), weak (0.10-0.39), moderate (0.40-0.69), strong (0.70-0.89), and very strong (0.90-1.00) [24]. Statistical significance was set at p<0.05. All analyses were performed using IBM SPSS Statistics for Windows, Version 29.0.1.0 (Released 2023; IBM Corp., Armonk, New York, United States).

## Results

All 130 recruited participants agreed to participate in the study and completed the data collection. Of these, 113 participants without missing data (aged 23-89 years, 51.3% women) were included in the analysis. A total of nine participants were excluded from the youngest group, four from the middle-aged group, and four from the oldest age group. Table 1 presents the sociodemographic and anthropometric characteristics of the participants, as well as their walking and physical activity behaviors assessed using the Japanese IPAQ-L and the ActiGraph.

**Table 1:** Sociodemographic and anthropometric characteristics and walking and physical activity behaviors of participants by age group (n=113). The table presents % (n) for sociodemographic attribute categories or median (25th, 75th percentile) for continuous data. ^a^No participant reported either "fair" or "poor". BMI: body mass index; Japanese IPAQ-L: Japanese version of the International Physical Activity Questionnaire long form; PA: physical activity

	All (n=113)	Age group		
		23-39 years (n=28)	40-64 years (n=48)	65-89 years (n=37)
Sociodemographic and anthropometric characteristics				
Gender				
% (n) men	48.7% (55)	57.1% (16)	43.8% (21)	48.6% (18)
Living area				
National Capital Region	47.8% (54)	25% (7)	22.9% (11)	97.3% (36)
Living arrangement				
% (n) living alone	27.4% (31)	25% (7)	20.8% (10)	37.8% (14)
Work status				
% (n) working full-time	60.2% (68)	96.4% (27)	81.3% (39)	5.4% (2)
% (n) working part-time	15% (17)	3.6% (1)	16.7% (8)	21.6% (8)
Perceived health^a^				
% (n) excellent	16.8% (19)	25% (7)	16.7% (8)	10.8% (4)
% (n) very good	43.4% (49)	32.1% (9)	47.9% (23)	45.9% (17)
% (n) good	39.8% (45)	42.9% (12)	35.4% (17)	43.2% (16)
Overweight/obese				
% (n) BMI ≥25 kg/m^2^	18.6% (21)	17.9% (5)	14.6% (7)	24.3% (9)
Walking and physical activity behaviors estimated by the Japanese IPAQ-L				
Total walking				
Median	154.0	145.0	85.0	270.0
(25th, 75th percentile), min/wk	(60.0, 322.0)	(45.0, 270.0)	(7.5, 290.0)	(135.0, 513.0)
Walking at work				
Median	0.0	20.0	0.0	0.0
(25th, 75th percentile), min/wk	(0.0, 45.0)	(0.0, 60.0)	(0.0, 38.8)	(0.0, 0.0)
Walking for transport				
Median	60.0	90.0	30.0	150.0
(25th, 75th percentile), min/wk	(0.0, 180.0)	(16.3, 157.5)	(0.0, 71.3)	(60.0, 235.5)
Walking for leisure				
Median	20.0	0.0	0.0	120.0
(25th, 75th percentile), min/wk	(0.0, 120.0)	(0.0, 60.0)	(0.0, 90.0)	(0.0, 275.0)
Moderate PA (not including walking)				
Median	150.0	80.0	90.0	280.0
(25th, 75th percentile), min/wk	(30.0, 410.0)	(12.5, 300.0)	(22.5, 325.0)	(105.0, 627.5)
Moderate PA+total walking				
Median	390.0	275.0	245.0	750.0
(25th, 75th percentile), min/wk	(170.0, 767.5)	(131.3, 477.5)	(120.0, 535.0)	(375.0, 915.0)
Physical activity behavior measured by the ActiGraph				
Moderate PA				
Median	200.0	234.0	198.5	176.0
(25th, 75th percentile), min/wk	(115.0, 309.0)	(170.5, 327.3)	(129.3, 325.0)	(87.0, 295.0)

The proportion of men and women was approximately equal across all age groups. No participants in any group rated their health as fair or poor. Notable differences were observed between the oldest age group (65-89 years) and the youngest to middle-aged groups (≤64 years) regarding living area and work status. While 97% of the oldest age group resided in the National Capital Region and only 27% were employed full- or part-time, 25% or fewer of the youngest to middle-aged groups lived in the National Capital Region, with over 81% working full-time. Additionally, participants in the oldest age group were more likely to live alone.

While the total time spent in moderate physical activity measured by the ActiGraph decreased with age, the total walking time assessed by the Japanese IPAQ-L was apparently the longest in the oldest age group. They spent more time particularly walking for transport and leisure according to the Japanese IPAQ-L. In contrast, more than half of the youngest to middle-aged groups reported no time spent walking for leisure at all (median: 0 min/week). Time spent walking at work was minimal across all age groups. Additionally, participants in the oldest age group reported the longest total time engaged in moderate physical activity (not including walking) and in the combined measure of moderate physical activity and walking. It is worth noting that 21 out of 113 participants reported no moderate physical activity (not shown in the table), emphasizing the substantial role of walking in their overall activity.

Table 2 shows Spearman's rank correlation coefficients (ρ) between Japanese IPAQ-L walking or moderate physical activity and ActiGraph moderate physical activity. Total walking showed weak to moderate correlations for all age groups (ρ=0.32 for the total group; ρ=0.38 for the youngest age group; ρ=0.35 for the middle-aged group; ρ=0.55 for the oldest age group). In contrast, correlations for moderate physical activity, whether or not walking was included, were negligible.

**Table 2:** Spearman's rank correlation coefficients (ρ) of Japanese IPAQ-L walking and moderate physical activity with ActiGraph moderate physical activity (min/wk) for the total sample and by age group (n=113). *p<0.05 Japanese IPAQ-L: Japanese version of the International Physical Activity Questionnaire long form; PA: physical activity

	All (n=113)		Age group					
			23-39 years (n=28)		40-64 years (n=48)		65-89 years (n=37)	
Japanese IPAQ-L	Correlation coefficient	P-value	Correlation coefficient	P-value	Correlation coefficient	P-value	Correlation coefficient	P-value
Total walking (min/wk)	0.32	<0.01*	0.38	<0.05*	0.35	0.02*	0.55	<0.01*
Walking at work (min/wk)	0.04	0.70	0.46	0.01*	−0.15	0.31	−0.11	0.53
Walking for transport (min/wk)	0.34	<0.01*	0.47	0.01*	0.47	<0.01*	0.32	0.05
Walking for leisure (min/wk)	0.28	<0.01*	−0.06	0.78	0.22	0.14	0.72	<0.01*
Moderate PA (not including walking; min/wk)	−0.13	0.16	−0.04	0.86	0.01	0.94	−0.30	0.08
Moderate PA+total walking (min/wk)	0.07	0.44	0.13	0.50	0.22	0.13	0.12	0.46

When walking was examined by domain, correlation strength and significance differed across age groups. Walking at work showed a moderate correlation only in the youngest age group (ρ=0.46), but not in other age groups. Walking for transport showed weak to moderate correlations in the total group (ρ=0.34), the youngest age group (ρ=0.47), and the middle-aged group (ρ=0.47), although it was not significant in the oldest age group (ρ=0.32; non-significant). Walking for leisure showed weak correlations in the total group (ρ=0.28) and a strong correlation in the oldest age group (ρ=0.72), but no significant correlation in the youngest and middle-aged groups (ρ=−0.06 and ρ=0.22, respectively; both non-significant).

## Discussion

This study aimed to evaluate the concurrent validity of the Japanese IPAQ-L for assessing walking behavior by comparing self-reported walking time with an accelerometer-derived measure of moderate physical activity in young to older adults. The results demonstrated weak to moderate correlations for total walking across all age groups, with the strongest association observed in the oldest age group (ρ=0.32 for the total group; ρ=0.38 for the youngest age group; ρ=0.35 for the middle-aged group; ρ=0.55 for the oldest age group). In contrast, no meaningful correlations were found for moderate physical activity, regardless of whether walking was included.

Our findings were largely consistent with those of a previous study of adults aged 18-84, which found that walking assessed by the IPAQ long form correlated with objectively measured moderate activity (ρ=0.27 for the total group; ρ=0.24 for the youngest age group; ρ=0.33 for the middle-aged group; ρ=0.29 for the oldest age group), although the correlation was not significant for the youngest age group in the previous study [15]. Also, this previous study reported stronger correlations between IPAQ's moderate physical activity and objectively measured light physical activity (ρ=0.37 for not including walking and ρ=0.34 for including walking, both for the total group), compared to objectively moderate physical activity (ρ=−0.12 for not including walking and ρ=0.03 for including walking, both for the total group) [15].

Despite differences in language versions, that is, Japanese in our study and German, French, and Italian in theirs [15], both studies yielded similar results, suggesting consistency across languages. These results may be attributable to the fact that accelerometers are particularly effective for capturing ambulatory activities, which constitute the majority of adults' habitual activity, but have limitations in detecting non-ambulatory activities such as cycling [25]. Also, it is also possible that self-reported moderate physical activity may not fully capture activities with moderate intensity as estimated by accelerometers, which could explain the weaker correlation observed in our study.

The variations in the correlation across age groups may be explained by differences in their walking behavior patterns and sociodemographic characteristics (Table 1). The strongest correlation for total walking in the oldest age group may reflect their higher engagement in transport- and leisure-related walking in their daily life, making self-reported recall more accurate. Meanwhile, work-related walking showed no significant correlation in middle-aged and older adults, likely due to their lower employment percentages and minimal reported time spent walking at work. For similar reasons, the lack of significant correlations for leisure-related walking in the youngest to middle-aged groups may be attributable to the limited time available for leisure walking [4], particularly on workdays, when leisure time is often scarce in these age groups.

This study has several strengths. It included a diverse sample spanning a wide age range, facilitated a comprehensive analysis of age-related differences in the validity of the Japanese IPAQ-L. In addition, the use of domain-specific walking categories provided insights into how different walking behaviors relate to accelerometer-based measures of physical activity. These strengths are particularly important when considering the applicability of the IPAQ-L to older adults because it enabled a detailed examination of walking behavior across different life stages.

Despite its strengths, this study has several limitations. First, the use of a convenience sample may limit the generalizability of the results to the broader Japanese population. The proportion of overweight or obese participants (18.6%) was lower than the reported rates in Japanese adults (31.5% for men and 21.1% for women) [26]. Moreover, our participants were willing to wear the ActiGraph for seven days, and none reported fair or poor health, suggesting that they may have been relatively healthy and health conscious. Considering these factors, the correlation may be weaker if the same survey is conducted in a less health-conscious population, as such individuals may have less distinct memories of their health-related behaviors in daily life. Second, while we examined self-reported walking behavior using the Japanese IPAQ-L, we did not assess walking speed or intensity, which could influence the relationship between self-reported and accelerometer-based measures. Third, the study relied on a single type of accelerometer (ActiGraph), which, despite being widely validated, might introduce device-specific biases. Future studies should include a more diverse sample and consider incorporating additional objective measures, such as Global Positioning System (GPS) tracking, to enhance the accuracy of walking behavior assessments, particularly for older adults, whose movement patterns may differ from those of younger adults.

## Conclusions

The Japanese IPAQ-L has weak to moderate concurrent validity for assessing total walking across age groups. Notably, the validity was higher in older adults. These findings suggest that the Japanese IPAQ-L can be used to rank individuals based on their total walking, with reasonable applicability to older adults. However, researchers should consider domain- and age-specific differences when interpreting results. Future studies could examine the validity of the questionnaire by using devices specifically designed to objectively assess walking behavior, such as GPS, which might provide more accurate evaluations.
